# Role and Value of Nitrogen Regulation Provided by Oysters (*Crassostrea virginica*) in the Mission-Aransas Estuary, Texas, USA

**DOI:** 10.1371/journal.pone.0065314

**Published:** 2013-06-06

**Authors:** Jennifer Beseres Pollack, David Yoskowitz, Hae-Cheol Kim, Paul A. Montagna

**Affiliations:** 1 Department of Life Sciences, Texas A&M University-Corpus Christi, Corpus Christi, Texas, United States of America; 2 Harte Research Institute, Texas A&M University-Corpus Christi, Corpus Christi, Texas, United States of America; 3 I.M. Systems Group at National Centers for Environmental Prediction, National Oceanic Atmospheric Administration, College Park, Maryland, United States of America; National Institute of Water & Atmospheric Research, New Zealand

## Abstract

Suspension-feeding activities of oysters impart a potentially significant benefit to estuarine ecosystems via reduction of water column nutrients, plankton and seston biomass, and primary productivity which can have a significant impact on human well-being. This study considered nitrogen regulation by eastern oysters *Crassostrea virginica* in the Mission-Aransas Estuary, Texas, USA, as a function of denitrification, burial, and physical transport from the system via harvest. Oyster reefs were estimated to remove 502.5 kg N km^−2^ through denitrification of biodeposits and 251.3 kg N km^−2^ in burial of biodeposits to sediments. Nitrogen is also physically transported out of the estuary via harvest of oysters. Commercial harvest of oysters in the Mission-Aransas Estuary can remove approximately 21,665 kg N per year via physical transport from the system. We developed a transferable method to value the service of nitrogen regulation by oysters, where the potential cost equivalent value of nitrogen regulation is quantified via cost estimates for a constructed biological nutrient removal (BNR) supplement to a wastewater treatment plant. The potential annual engineered cost equivalent of the service of nitrogen regulation and removal provided by reefs in the Mission-Aransas Estuary is $293,993 yr^−1^. Monetizing ecosystem services can help increase awareness at the stakeholder level of the importance of oysters beyond commercial fishery values alone.

## Introduction

Oysters have long been acknowledged for their economic importance as a commercially and recreationally harvested species. Within the United States, Texas produces the second-largest oyster harvest, with an estimated $19 million generated in 2010 [1]. Additionally, oysters offer a suite of critically important ecosystem services that benefit human well-being. Oyster reef structures provide essential refuge and foraging habitat for fish [2]–[4] and invertebrate species [5]. Filter feeding imparts a potentially tremendous benefit to the ecosystem by influencing nutrient cycling, gas regulation, and water quality [6]–[8]. Furthermore, the presence of reefs can positively impact the growth of submerged aquatic vegetation (SAV) either through removing suspended solids or acting as a breakwater to reduce sediment re-suspension, both which increase light penetration [9], [10].

Shellfish (including oyster) reefs, are the most imperiled marine habitats on earth, with an estimated 85% lost in relation to historic levels [11]. In response to the drastic decline in oyster populations, there has been an increased focus on the loss of associated ecosystem services that contribute to human well-being (Table 1) [12]–[15]. Suspension feeding and nutrient regulation activities of oysters have been of particular interest, as they can ameliorate some of the negative effects of nutrient overenrichment, including enhanced primary production and algal blooms [16]. Using a dynamic ecosystem model, Cerco and Noel [17] estimated water quality improvements resulting from different oyster biomass scenarios in Chesapeake Bay. Results suggested that a ten-fold increase in oyster biomass could potentially remove 30,000 kg d^−1^ nitrogen from the system. Newell et al. [18] estimated the value of oyster-mediated nitrogen removal of 13,080 kg N yr^−1^ from the upper Choptank estuary at $314,836. Using an average value for nitrogen removal, Kasperski and Wieland [19] estimated an ecosystem service value of $18,135.69 per million oysters in Chesapeake Bay.

**Table 1:** Ecosystem services of oysters, using functions and services modified from Faber et al. (2006).

Service	Description	Oyster Example
**Habitat**	Physical place where organisms reside	Fish and invertebrate habitat
**Gas Regulation**	Regulation of the chemical composition of the atmosphereand oceans	Potential sequestration of atmospheric carbon dioxide
**Disturbance Regulation**	Dampening environmental fluctuations and disturbance	Shoreline protection, buffering wave energies, erosion prevention
**Water Regulation**	Flow and purification of water	Influences circulation patterns
**Soil Retention**	Erosion control and sediment retention	Sediment stabilization, creation of shell hash and sand as shells break down
**Nutrient Regulation**	Maintenance of major nutrients within acceptable bounds	Reduce water column nutrients, phytoplankton and primary productivity; clean water
**Food**	Provisioning of edible plants and animals for human consumption	Commercial and subsistence harvesting
**Raw materials**	Building, manufacturing, fuel, soil, fertilizer	Road base, chicken calcium supplement, cosmetics
**Ornamental Resources**	Resources for fashion, handicraft, jewelry, etc.	Belt buckles, ornamental construction
**Recreation**	Opportunities for rest, refreshment, recreation	Fishing, birdwatching
**Science and Education**	Use of natural areas for science and educationalenhancement	Research about oysters, natural reefs provide metrics for restoration
**Spiritual and historic**	Spiritual and historical information	Middens, oystermen, seafood festivals

The goal of this study was to build on previous research, primarily from the Atlantic U.S. coast, to estimate the role and value of nutrient regulation provided by eastern oysters within a Gulf of Mexico estuary using the replacement cost method. Quantitative valuation of ecosystem services provided by oysters is important for increasing awareness of stakeholders, and is particularly critical for justifying restoration and recovery dollars post-disturbance that exceed the calculated value as a fishery.

## Methods

### Study Location

**Figure 1:**
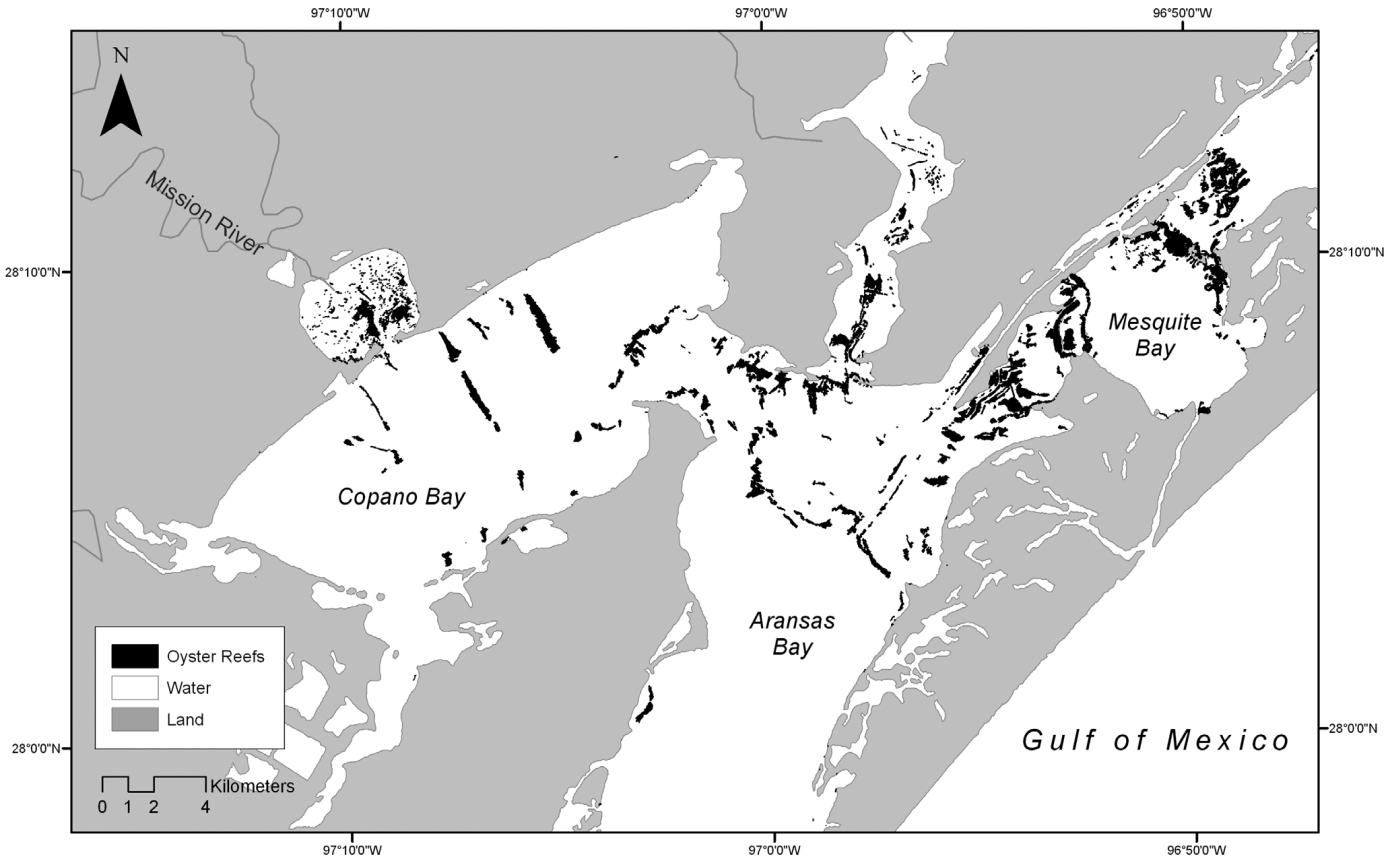
Mission-Aransas Estuary, Texas, USA, showing locations of oyster reefs.

Our study site is the Mission-Aransas estuary, Texas, USA. The shallow, bar-built estuary is approximately 540 km^2^ with an average depth of 2 m at mid-tide level (Figure 1) [20]. The estuary comprises several bays, the largest of which are Copano Bay, Aransas Bay and Mesquite Bay. Oysters occur primarily as large subtidal reefs in low- to moderate-salinity regions of the estuary [21]. Vertical relief of the reefs ranges from ∼0.3 to 1.8 m in height, and the areal extent of is approximately 18.11 km^2^ (1811 ha).

### Field Data

Oysters and environmental variables were collected monthly throughout 2007 and 2008 by oyster dredge at randomly selected locations on known reefs in Copano Bay and Aransas Bay in cooperation with the Texas Parks and Wildlife Department’s (TPWD) Coastal Fisheries Division as described [22]. All necessary collecting permits were obtained from TPWD. Up to 20 live oysters per location were randomly selected and measured for shell height and biomass parameters (wet weight, dry weight, ash-free dry weight). Water samples were collected and hydrographic characteristics (salinity, temperature, dissolved oxygen, pH) were measured approximately 0.2 m above the top of the oyster reef using a YSI series 6 data sonde. Chlorophyll-*a* (Chl-*a*) samples were filtered onto glass fiber filters and placed on ice (<4.0°C). Chl-*a* was extracted overnight and read fluorometrically on a Turner Model 10-AU using a non-acidification technique (EPA method 445.0) [23]. Total suspended solids were measured using EPA method 160.2.

### Nitrogen Regulation by Oysters

Nitrogen can be removed from an estuary via physical transport, denitrification, or burial [24]. Bivalves remove significant amounts of planktonic nitrogen from the water column, which is assimilated at rates that vary seasonally [25]. Eastern oysters can assimilate an estimated 50% of the particulate organic nitrogen (PON) filtered, with the remainder voided as biodeposits [26]. Assimilated nitrogen can be removed from the estuarine system via oyster harvest or temporarily sequestered in shell and tissue and available to tertiary consumers. Oyster biodeposits may undergo burial and/or denitrification [18], also resulting in nitrogen removal from the system [27], [28] [29].

Seasonal oyster filtration rates were empirically derived [30] using the general form:
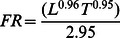
(1)where *FR* is filtration rate (ml ind^−1^ min^−1^), *L* is oyster shell height (mm), and *T* is temperature (°C).

This equation for filtration rate is modified as a function of salinity [29]:
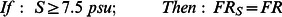
(2)

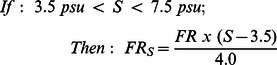
(3)

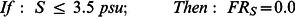
(4)where *S* is salinity and *FR_S_* is the salinity effect-corrected filtration rate (ml ind^−1^ min^−1^).

Filtration rate is also modified as a function of suspended solids in the water column [30] as:
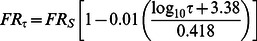
(5)where τ is total particulate content (g l^−1^).

Seasons were designated as follows: winter = Dec, Jan, Feb; spring = Mar, Apr, May; summer = Jun, Jul, Aug; fall = Sep, Oct, Nov.

The amount of food available to oyster populations is a function of the amount of food present and the characteristics of water flow delivering the food particles [31], [32]. Chlorophyll-*a* (Chl-*a*) concentration can be used as an index of available food [33], [32] using:
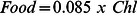
(6)where *Chl* is Chl-*a* concentration in µg l^−1^. Chl-*a* was then converted to nitrogen using an established Chl-*a*:N ratio from the literature [34].

Nitrogen uptake is a function of clearance rate and assimilation efficiency. Seasonal clearance rates were then calculated as [30]:
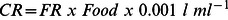
(7)where *CR* is clearance rate (µg N ind^−1^ min^−1^), *FR* is filtration rate (ml ind^−1^ min^−1^), and *Food* is available food converted from Chl-*a* in nitrogen units (µg N l^−1^).

Existing field and laboratory measurements were used to estimate nitrogen removal. To determine the amount of undigested PON that was transferred to sediments in biodeposits, an average nitrogen assimilation efficiency of 50% was applied, as derived from physiological data for eastern oysters [26]. We estimated the amount of PON from biodeposits that is removed through coupled nitrification-denitrification by applying a denitrification rate of 20%, from laboratory derived estimates for eastern oysters [27]. We also applied an average nitrogen burial rate of 10% to the seasonal biodeposition data, based on field measurements from the Choptank River [29]. This method and series of assumptions follow those used by Newell et al. [18] to derive the 33% annual loss estimated for natural intertidal oyster reefs [35].

In order to make our estimates comparable to other systems, we scaled up from individual oyster physiology to standardized units of kg N km^−2^ yr^−1^. We first calculated nitrogen regulation per km^2^ in the Mission-Aransas estuary per year by applying the average number of oysters (408 m^−2^) and the areal extent of oyster reefs within the estuary (18.11 km^2^). A 25% live-to-dead ratio was applied (Beseres Pollack, unpublished data), to adjust for dead oysters that are not producing biodeposits.

### Valuation

A straight forward approach to assessing the cost equivalent value of the nutrient regulating service that oysters provide with regards to nitrogen, is to look at the replacement costs to provide an engineered solution via wastewater treatment and specifically a biological nitrogen removal (BNR) processes. The engineered BNR process removes total nitrogen and total phosphorus from wastewater through the use of microorganisms under different environmental conditions [36].

Replacement costs can be a suitable measure of economic value if three conditions are met: i) the engineered system provides the same service as the natural system, ii) the alternative is the least cost, and iii) the services are demanded by society [37]. We estimated the amount of nitrogen removed through an engineered process using the Back River Wastewater Treatment Plant in Maryland as the processing example of the BNR method [36]. The Back River plant, which can process 180 million-gallons-day (mgd), was used because it had relevant data available appropriate to demonstrate the method described here. The dollar cost equivalent of nitrogen regulation via an engineered process was calculated given capital, maintenance, and operation costs for the entire Mission-Aransas estuary.

## Results

**Figure 2:**
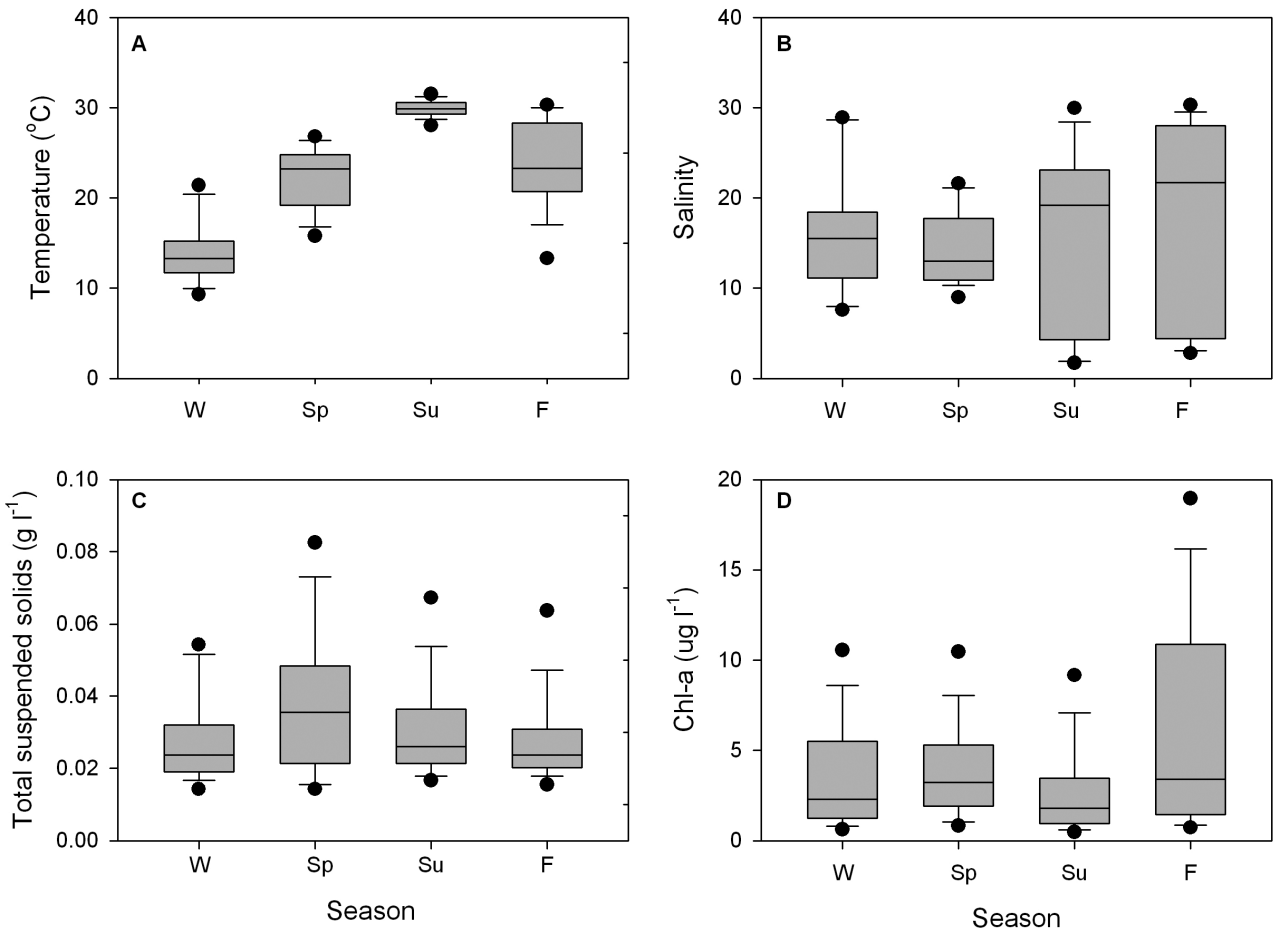
Seasonal environmental variables measured in the Mission-Aransas Estuary, Texas. (W = winter, Sp = spring, Su = summer, F = fall).

Environmental conditions were variable within the Mission-Aransas Estuary (Figure 2). Seasonal temperatures ranged from (mean ± SD) 14.4±3.4°C in winter to 30.4±1.0°C in summer. Salinities ranged from (mean ± SD) 15.1±8.4 in winter to 29.6±6.8 in summer. Total suspended solids ranged from (mean ± SD) 0.0277±0.02 g l^−1^ in winter to 0.0365±0.02 g l^−1^ in spring. Seasonal Chl-*a* concentrations ranged from (mean ± SD) 2.69±2.60 ug l^−1^ in summer to 6.31±6.01 ug l^−1^ in fall. Average oyster sizes (shell heights, ± SD) were 69.6±21.7 mm in winter, 73.8±20.8 mm in spring, 70.6±22.3 mm in summer, and 62.5±23.3 mm in fall.

Average oyster filtration rates were calculated seasonally for the Mission-Aransas estuary and ranged from (mean ± SD) 14.5±5.2 ml ind^−1^ min^−1^ in winter to 23.52±7.53 ml ind^−1^ min^−1^ in spring (Table 2). After adjusting for food availability, Chl-*a* was converted to nitrogen at 1 µg Chl-*a*:14 µg N [34]. Seasonal clearance rates were then calculated, ranging from (mean ± SD) 0.063±0.02 µg ind^−1^ min^−1^ in winter to 0.124±0.09 µg ind^−1^ min^−1^ in fall (Table 2).

**Table 2:** Monthly average water temperature (°C), seston concentrations (measured as TSS (mg L^−1^)), and phytoplankton Chl-*a* (µg L^−1^) in the Mission Aransas Estuary.

					Seasonal nutrient removal	
	Water temp (°C)	Seston(mg L^−1^)	Chl a(µg L^−1^)	Clearance rate (µg ind^−1^ min^−1^)	kg N denitrified km^−2^	kg N buried km^−2^
**Winter**	14.4	27.7	3.68	0.063	85.20	42.60
**Spring**	20.7	36.5	4.12	0.115	154.71	77.36
**Summer**	30.4	29.8	2.69	0.072	96.86	48.43
**Fall**	23.1	28.4	6.31	0.124	165.73	82.87
**Annual Total**					502.50	251.25

See text for details of calculations of clearance rate and N removal.

Oysters assimilate nitrogen with a wide range of efficiencies, due in part to sources of particulate organic matter [38] and season [39]. We applied an average assimilation efficiency of 50% as reported [26] for *Crassostrea virginica* feeding on natural seston at comparable concentrations (4 to 20 mg L^−1^) in order to estimate the total amount of undigested PON transferred to the sediments in biodeposits. Scaling up to seasonal and annual averages, nitrogen removal from biodeposits associated with coupled nitrification-denitrification ranged from 85.2 kg N km^−2^ in winter to 165.7 kg N km^−2^ in fall, with an annual total of 502.5 kg N km^−2^, or 9100.28 kg N for 18.11 km^2^ of oyster reef in the Mission-Aransas Estuary. Seasonal average nitrogen removal due to PON in biodeposits that is buried in sediments ranged from 42.6 kg N km^−2^ in winter to 82.9 kg N km^−2^ in fall, with an annual total of 251.3 kg N km^−2^ or 4550.14 kg N for 18.11 km^2^ of oyster reef in the Mission-Aransas Estuary.

Nitrogen is also physically transported out of the estuary via harvest of oysters. Approximately 8,497,910 kg of oysters (wet meat weight 450,647 kg) were harvested in 2007 and 3,862,997 kg of oysters (wet meat weight 204,856 kg) were harvested in 2008 from the Mission-Aransas Estuary (TPWD commercial landings data). Eastern oyster tissue and shell both comprise nitrogen – at approximately 7% and 0.3% respectively on a dry weight basis [16] – which can be permanently removed from the system as a result of harvest. The allometric relationship between wet and dry weight for oysters in the Mission-Aransas Estuary [22] was then applied:
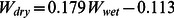
(8)where *W_dry_* and *W_wet_* are dry weight (g) and wet weight (g) of oyster tissue, respectively. Applying this relationship, approximately 80,666 kg of tissue and 36,669 kg of tissue (on a dry-weight basis) were harvested in 2007 and 2008, respectively. Therefore, approximately 29,789 kg N was removed at harvest in 2007, and 13,541 kg N was removed at harvest in 2008.

How much total nitrogen is removed through an engineered process depends upon the influent and technology used to remove it. At the Back River Wastewater Treatment Plant in Maryland, which uses the Modified Ludzack-Ettinger process (MLE), influent into the BNR process contains 30 mg/l total nitrogen [36]. After treatment, it is 7.6 mg/l, close to a 75% reduction or 22.4 mg/l. Therefore, a facility like the Back River Wastewater Treatment Plant theoretically could remove:
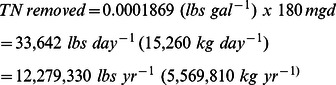
(9)


Capital, and maintenance and operations costs vary with the type of technology employed as well as the design capacity of the plant. We have calculated that the oyster reefs in the Mission-Aransas system can remove approximately 21,665 kg N per year (average 2007–2008) via physical transport from the system through harvest. This permanent removal equates to ∼0.4% of the of the Back River BNR facility while the denitrification and burial equates to ∼0.25% of Back River. Therefore, oysters that are harvested can provide the nutrient regulation service, removal in this case through harvest, roughly equivalent to the size of a 0.7 mgd wastewater treatment plant, which has a calculated capital cost of:
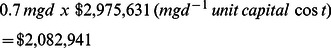
(10)


Where the unit capital cost is calculated from the relevant MLE plants in [36]. The annualized value based upon a 15 year life span of a BNR system [40] straight-line depreciation schedule with no scrap value would be $138,863. Straight-line depreciation is the expensing of the original cost of the asset in equal increments over the useful life of the asset. In our example this technique is used to allocate the cost equivalent for each year: $2,082,941/15 yrs = $138,863. Assuming conservative maintenance and operation expenses of 2% cost of capital annually [41], the potential annual engineered cost equivalent of the service of nitrogen regulation and removal provided by harvest of oysters in the Mission-Aransas system is:
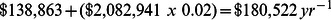
(11)


Utilizing the same approach described above, the capital cost equivalent for nitrogen removal via:

Denitrification.
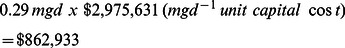
(12)


Burial.
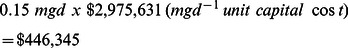
(13)


The annualized cost of a BNR system, once again assuming a 15 year life span and no scrap value, is $57,529 for denitrification and $29,756 for burial. With an annual maintenance and operation cost 2% cost of capital the engineered cost equivalent of the nitrogen regulation and removal service is:

Denitrification
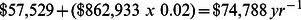
(14)


Burial
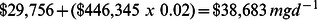
(15)


The combined value from oysters in place, aiding in denitrification and burial, is $113,471 *yr* ^−*1*^ (see Table 3).

**Table 3:** Nitrogen removal and cost equivalent values provided by 18.11 km^2^ of oyster reef in the Mission-Aransas Estuary.

	Harvest	Denitrification	Burial	Total
**Annual nitrogen removal (kg)**	21,665	9,100	4,550	35,315
**Cost equivalent value (** ***yr*** ^−***1***^ ***)***	$180,522	$74,788	$38,683	**$293,993**

## Discussion

Nutrient overenrichment of coastal areas is an ongoing global issue. The most common strategy for dealing with eutrophication is reduction of anthropogenic nutrient loads [42], [43]. Habitat restoration has also been suggested as a potential mechanism for improved nitrogen removal [17], [44], [45]. Suspension feeding activities of oysters can improve water quality in estuarine systems by exerting top-down control on phytoplankton populations [46], [16]. Eastern oysters are unique among other suspension-feeding bivalves in that they can maintain high clearance rates even at high seston concentrations [18]. Oysters transfer water column nitrogen to the sediments in biodeposits, enhancing removal via burial and denitrification [18], [28], [47]. Oysters can also sequester and assimilate nitrogen from the water column into shell and tissue, which can be removed from the system via direct harvest.

We calculated that the 18.11 km^2^ oyster reefs in the Mission-Aransas Estuary can annually remove 9100 kg N via coupled denitrification on biodeposits and 4550 kg N via burial of biodeposits in sediments. We also calculated that harvested oysters in the Mission-Aransas system can remove approximately 21,665 kg N annually via physical transport from the system. Although harvested oysters can act as a vehicle for one-time permanent transport of N away from the estuary, unharvested oysters continue to provide nutrient regulation services over their life span [18]. The loss of filtration capacity concurrent with oyster population declines in the Chesapeake Bay over the past century [48] provides a particularly compelling illustration of this point.

The importance of nitrogen regulation services provided by oyster reefs has been demonstrated in several studies. Oyster restoration has been proposed as a management strategy for improving water quality in Chesapeake Bay via top-down control of phytoplankton populations, with estimates of approximately 753 kg N yr^−1^ removed per million oysters [18]. Use of habitat restoration to increase oyster biomass 10-fold in Chesapeake Bay is predicted to reduce Chl-*a* concentrations by 1 mg m^−3^ and remove 30,000 kg N d^−1^ through denitrification [17]. Denitrification rates commonly show seasonal differences, with higher rates in warmer months, concurrent with higher metabolism [49], [50], [28], [47]. Piehler and Smyth [28] measured denitrification rates in Bogue Sound, NC, and estimated the annual value of nitrogen removal by oysters as approximately $3,000 per acre per year. The amount of nitrogen pollution removed by oysters in the Choptank River would otherwise cost over $300,000 yr^−1^ to remove via combination of methods [18]. In order to have a meaningful effect, the population of oysters needs to be large enough to both remove new nitrogen being introduced to the system while also decreasing the existing concentration of nitrogen within the estuary [19].

Commercial and recreational harvest of oysters and other bivalves and extractive aquaculture provide direct mechanisms for nitrogen removal from coastal systems. Commercial mussel farming in Sweden has been predicted to reduce the net transport of nitrogen at the mouth of the Gullmar Fjord by 20%, providing a significant service to Swedish society [51]. The potential harvest of 10^6^ cultured oysters yr^−1^ from Chesapeake Bay is estimated to remove 132 kg N yr^−1^, and could offer financial incentives in the form of nutrient reduction payments to help scale-up aquaculture production activities [52], [53].

There is a clear trade-off between the one-time value of harvesting oysters and leaving them within the estuary to provide ongoing ecosystem services [18]. For example, in Chesapeake and Delaware Bays, severe declines in eastern oyster populations, due in part to habitat destruction and overfishing, corresponded with rapid ecosystem change to planktonic food-web dominant systems [48], [54]. When oysters are harvested, it potentially diminishes the pool of available suspension feeders and reduces their overall capacity to filter the water column [48].

Nutrient regulation is not the only service potentially supplied by oyster reefs. In fact, as Table 1 illustrates there are numerous services that only add to the value of nutrient regulation, so any monetary estimate that focuses only on one ecosystem service will undervalue a reef. For example, three-dimensional oyster reef structures provide nursery, refuge, and foraging habitat for fish and macroinvertebrates [2], [3]. Reefs can also provide sediment stabilization and shoreline protection from both human (wake from passing boats) and natural disturbance (storms) [55]. Cultural services such as recreational fishing over and around reefs [56], [57], science and education opportunities that are available when research is conducted on oysters [7], [18], food [58], and spiritual and historic services such as the tradition of oystermen [59] are also extremely important and valued by society.

In the current study, we demonstrate a process in which one ecosystem service can be monetized. The value is derived via a cost equivalent of an engineered approach using a BNR process. This ecosystem service is provided at no direct charge to the public but can have a significant impact on human well-being. For example, humans do not need to pay increased tax or utility rates for a wastewater treatment system that would include nutrient removal. When utilizing the replacement cost approach it should be done under the notion that the “costs” will not necessarily equate to “benefits” except under very restricted circumstances [60]. What is demonstrated here is simply a cost equivalent of the service based upon the BNR process.

While there is currently no regulatory need for nutrient regulation in the Mission-Aransas estuary, this pilot study demonstrates a valuation process that can be transferable to nutrient enriched systems. In eutrophic estuaries, oyster restoration is recommended as a complement, not a substitute, to load reduction [17]. The results of this study are sensitive to the assimilation rate and the capital cost. The assimilation rate varies based upon oyster size, available food, and environmental conditions [61]. The capital costs utilized in this study were the best available estimations on average and are not site specific, which would produce better estimates of the replacement cost value of nitrogen regulation. This study utilizes a novel approach, the replacement cost method, for estimating the ecosystem service of nutrient regulation provided by oysters. Previous studies most commonly associated with waste or nutrient regulation and storm protection are linked to the services provided by wetlands [62]–[64].

Other techniques exist for estimating the value of ecosystem services such as willingness-to-pay (WTP), attribute based stated-choice, hedonic pricing, and travel cost. Utilizing different techniques will generate different values so it is critical to employ the appropriate technique for the question being asked. For example, Piehler and Smyth [28] utilized values derived from the North Carolina nutrient offset trading program of $13 per kg of nitrogen removed in order to quantify the denitrification value of various estuarine habitats. Newell et al. [18] employ an average monetary value of $24.07 kg ^−1^ for removing nitrogen from an early study and apply it specifically to their study site. Including the combined value for denitrification and burial calculated in this study of $8.33 kg^−1^ using a replacement cost approach, the values generated by these three studies are reasonably similar given their different approaches.

Effective management of oyster reefs and the services they provide requires that both natural and social scientists collaborate to develop tools that can be utilized by stakeholders. Monetizing ecosystem services can help increase awareness at the stakeholder level of the importance of oysters beyond commercial fishery values alone. While discussion of potential reef conservation and restoration mechanisms are beyond the scope of this paper, serious consideration should be given to the process of “operationalizing” ecosystem services of oyster reefs for restoration so that both public and private entities have the opportunity to support restoration and at the same time receive credit for enhanced services, such as reducing nutrient loads, creating habitat, or protecting shorelines. An increase in a multidisciplinary effort, including funding, to quantify coastal and marine ecosystem services both in monetary and non-monetary terms is critical to help close the information gap that exists to help make effective management decisions.
